# Serological survey in a university community after the fourth wave of COVID-19 in Senegal

**DOI:** 10.1371/journal.pone.0298509

**Published:** 2024-11-21

**Authors:** Fatou THIAM, Abou Abdallah Malick DIOUARA, Clemence Stephanie Chloe Anoumba NDIAYE, Ibrahima DIOUF, Khadim KEBE, Assane SENGHOR, Djibaba DJOUMOI, Mame Ndew MBAYE, Idy DIOP, Sarbanding SANE, Seynabou COUNDOUL, Sophie Deli TENE, Mamadou DIOP, Abdou Lahat DIENG, Mamadou NDIAYE, Saidou Moustapha SALL, Massamba DIOUF, Cheikh Momar NGUER

**Affiliations:** 1 Groupe de Recherche Biotechnologies Appliquées & Bioprocédés Environnementaux, École Supérieure Polytechnique, Université Cheikh Anta Diop de Dakar, Dakar, Senegal; 2 Laboratoire Physique de l’Atmosphère et de l’Océan-Siméon Fongang, École Superieure Polytechnique, Université Cheikh Anta Diop de Dakar, Dakar, Senegal; 3 Laboratoire d’Imagerie Médicale et de Bio-Informatique, École Superieure Polytechnique, Université Cheikh Anta Diop de Dakar, Dakar, Senegal; 4 Laboratoire Mathématiques Appliquées et Informatique, Faculté des Sciences et Techniques, Université Cheikh Anta Diop de Dakar, Dakar, Senegal; 5 Laboratoire Sante Publique, Institut d’odontologie et de Stomatologie, Faculte de Medecine, de Pharmacie et d’Odonthologie, Université Cheikh Anta Diop de Dakar, Dakar, Senegal; Instituto Nacional de Salud Pública: Instituto Nacional de Salud Publica, MEXICO

## Abstract

A cross-sectional survey was conducted at Polytechnic High School (PHS) to assess the spread of COVID-19 infection among students and staff. A random cluster sampling was conducted between May 19 and August 18, 2022, after the fourth wave of COVID-19 in Senegal. IgM and IgG SARS-CoV-2 antibodies were screened using WANTAI SARS-CoV-2 ELISA assays. Seroprevalence and descriptive statistics were calculated, and associations between seropositivity and different factors were determined using logistic regression. A total of 637 participants were recruited and the median age was 21 years [18–63]. 62.0% of the participants were female, and 36.89% were male, with a male-to-female ratio = 0.59. The overall IgG and IgM seroprevalence were 92% and 6.91% respectively. Among those who tested positive for IgM, 6.75% were also positive for IgG, and 0.15% were negative for IgG. Interestingly, 6.90% of participants tested negative for both IgM and IgG. We found a higher IgM seroprevalence in men than women (9.4% vs. 5.6%) and a lower IgM seroprevalence in (18–25) age group compared to (55–65) years. We revealed a significant difference according to IgG seroprevalence among participants who declared fatigue symptoms [92.06% (95% CI: 89.96–94.16)] compared to those who did not [80.39% (95% CI: 77.31–83.47)], *p* = 0.0027. IgM seropositivity was found to be associated with Body Mass Index (BMI) categories (O.R. 0.238, p = 0.043), ethnic group (O.R. 0.723, *p* = 0.046), and marital status (O.R. 2.399, *p* = 0.021). Additionally, IgG seropositivity was linked to vaccination status (O.R. 4.741, *p* < 0.001). Our study found that most students and staff at PHS were exposed to SARS-CoV-2, confirming the virus’s circulation at the time of the survey. We also identified differences in individual susceptibility that need further clarification. Our results highlight the importance of seroepidemiological surveys to assess the true impact of the COVID-19 pandemic in a community and to monitor variations in antibody response.

## Introduction

The Coronavirus Disease 2019 (COVID-19) was first identified in December 2019 in Wuhan, China. It is an infectious disease caused by the Severe Acute Respiratory Syndrome Coronavirus 2 (SARS-CoV-2) [1, 2]. On January 30 2020, the World Health Organization (WHO) declared COVID-19 a public health emergency of global concern, and on March 11, 2020, it announced the COVID-19 epidemic as a pandemic [3]. By November 12, 2023, more than 697 million people had been infected worldwide, with over 6.9 million deaths [4]. The COVID-19 outbreak had substantial economic, social, and health repercussions and damaging educational consequences [5–7]. In April 2020, the World Bank estimated that higher education institutions had been closed in 175 countries and that studies had been interrupted or significantly disrupted due to COVID-19, affecting more than 220 million students [8]. The pandemic forced academic communities to adopt online platforms for the continuity of teaching and learning activities, negatively impacting learning outcomes, particularly in developing countries where the lack of network infrastructures, computers, and internet access is challenging distance learning in developing countries [9, 10].

In Senegal, a West African country, the first case of SARS-CoV-2 was identified on March 2, 2020. Since then, the number of cases has risen considerably, and the country currently has more than 89,022 confirmed cases of COVID-19 and 1,971 deaths [11]. The onset of the pandemic led the government authorities to close higher education establishments from March 16 to August 31 2020. The establishments reopened with the implementation of sanitary protocols that emphasized the reinforcement of barrier measures. These measures included wearing masks, washing hands with soapy water or using hand sanitiser, maintaining social distancing, and taking body temperature at campus access points [12]. However, these measures have often been insufficient to stem the spread of the virus. Clusters quickly appeared in some establishments, including the Cheikh Anta Diop (CAD) University, located in Dakar, the capital of Senegal.

The failure of strategies to control the virus spread could be related to various factors, including the CAD University ecosystem, which accommodates over 80,000 students from diverse origins. The consequence is overcrowding in student residences that caused the virus spread; (ii) the university environment is mainly populated by young people, who do not seem to be fully aware of the risks associated with the COVID-19 health crisis [13]; (iii) the promiscuity in the Halls of residence, making it difficult to comply with social preventive measures; (iv) Lastly, lack of vaccine deployment and reluctance was noted among young people, which also contributes to low vaccination coverage in universities [14].

Faced with all these difficulties, it was essential to adopt cyclical measures to manage the COVID-19 pandemic, characterised by the virus spreading in waves [15, 16]. These measures could involve introducing survey studies based on diagnostic testing and mass screening protocols to diagnose and follow up with people exposed to or infected with SARS-CoV-2. In practice, detecting viral RNA by RT-PCR is the reference method for confirming the diagnosis of SARS-CoV-2 infection [17, 18]. However, access to diagnostic tests still needs to be improved in poor countries [19]. The other way to estimate the true extent of the epidemic is to conduct Seroprevalence surveys [20, 21]. Seroepidemiological studies to detect the presence of anti-SARS-CoV-2 antibodies are a valuable tool for assessing the timing of the epidemic. It can help to confirm the presence of a recent infection when PCR is limited. Some antibodies, such as IgG, can even be detected years after exposure [22]. Their detection can be used for tracking the spread of infection and defining herd immunity barrier and individual immunization levels in the ongoing COVID-19 pandemic [23]. By carrying out this analysis on a representative population, it is possible to estimate what proportion of the population has already been exposed to the new coronavirus [24]. This information helps identify the epidemic phases and can help the authorities make decisions and even anticipate appropriate measures to contain the pandemic’s spread [25]. Sero-epidemiological studies have been conducted in academic institutions such as universities in many countries [26–29]. However, more studies are highly relevant because university communities (faculty, staff and students) could be among the most exposed to SARS-CoV-2. In Senegal, no seroprevalence studies have been conducted in cohorts of educational institutions.

We conducted an on-site screening project at Polytechnic High School (PHS), a public institution with an inter-African focus at CAD University, from May to August 2022, following the fourth wave of the Coronavirus disease in Senegal. Our goal was to assess the true extent of previous and recent COVID-19 exposure among students and staff and investigate the risk factors associated with SARS-CoV-2 IgM and IgG seropositivity.

## Materials and methods

### Study design and population

The SARSESP (“Etude de Seroprevalence du SARS-CoV-2 au sein de l’École Su-périeure Polytechnique”, in the Cheikh anta DIOP University (CAD University)) project is an on-site university population-based cross-sectional study. The sampling occurred at the PHS, an establishment of CAD University. Students, Professors, and Technicians, Administrative and Service (TAS) officers at SPS were invited by e-mail to enrol in the study. Participants were volunteers who registered online between 19 May and 18 August 2022. A questionnaire was administered to each participant after consent, blood samples were taken for SARS-CoV-2 antibody detection.

### Sample size calculation

We used stratified random sampling: the first stratum concerned professors, the second TAS officers and the third students. Systematic random sampling was used within each stratum to determine the required subjects number. Using the lists provided by the student affairs and human resources departments, we calculated the sample size based on the hypothesis of an expected seroprevalence of 45%, with a precision of 5%, a design effect of 1.96, and a nonresponse rate of 65%. We determined that >450 participants needed to be recruited. To increase the accuracy of the results, this size was multiplied by 2, giving a sample size of 898, and rounded up to 1000 to account for any lost records. The survey step for selecting statistical units was 6133/1000, i.e. a step = 6. In this study, an allocation proportional to the size of each stratum was used. Thus, we considered a proportion of 83.54% for students, 9.97% for professors and 6.47% for TAS officers. Then, we obtained 65 TAS officers, 100 professors and 835 students. The inclusion criteria were age over eighteen (18) years, informed consent signed, and questionnaire completion. Then, the non-inclusion criteria were under 18 years of age, non-consent and contraindications to venous blood sampling (anaemia, Infection or hematoma at a prospective venipuncture site, etc.).

### Questionnaire

We shared an interviewer-administered questionnaire with participants on an electronic tablet (https://enquete.ucad.sn/index.php/598685?lang=fr). The questionnaire covered points regarding different factors to assess for relationships between IgM and IgG seropositivity and these factors. Questions relating to sociodemographic characteristics were collected, such as age, sex, occupation, education level, nationality, ethnic group and accommodation type. We also collected alcohol and tobacco intake, SARS-CoV-2 vaccination status, and preventive measures related to SARS–CoV–2 practices. We provided all recruitment participants with face masks and hand sanitisers and encouraged them to practice physical and social distancing. The questionnaire on COVID-19 was posted online by the IT and Information Systems Department of CAD University.

### Blood collection and SARS-CoV-2 antibodies detection

After each participant signed the written consent form, a 10 Ml whole blood sample was collected into a dry vacutainer tube by standard venipuncture technique. Blood samples were centrifuged at 2,500 rpm for 10 minutes. Then, the plasma was collected and stored in cryotubes at -80°C at the GRBA-BE laboratory biobank in PHS until the tests were carried out. Seropositivity to anti-SARS-CoV-2 antibodies was used as a biomarker of exposure to the SARS-CoV-2 virus. IgM antibodies emerge early during immune responses (usually from days 5–7 after symptoms appear, but sometimes later and decrease at days 15–22), while IgG antibodies typically appear later (detectable from day 11 post symptom, reaching a maximum 3–4 weeks after) and exist in human bodies for months [23, 30, 31].

Serological tests were performed by qualitative ELISA for IgM and quantitative ELISA for IgG following the instructions for the WANTAI SARS-CoV-2 IgM ELISA (Beijing Wantai Biological Pharmacy Enterprise, Beijing, China; Ref. WS-1196 and WS-1396) recommended by the WHO for seroepidemiological studies, which detects total antibodies (including IgM and IgG) binding the SARS-CoV-2 spike protein receptor binding domain (S1/RBD) [32]. Serum samples were analysed in duplicate according to the supplier’s recommendations.

### Statistical analyses

Statistical analyses were performed using Rstudio (version R 4.2.1) and GraphPad Prism (version 10.1.1) softwares. Continuous variables were described as mean (standard deviation) or median (interquartile range). Normally distributed variables were compared with a t-test, and nonparametric data were compared with the Mann-Whitney test. Categorical variables were presented as per cent, and Fisher exact tests or chi-squared tests were used for proportional assessments. For all statistical tests, we accepted a two-sided level of significance was set at p ≤ 0.05.

SARS-CoV-2 seroprevalence was defined as the ratio of the number of people who developed anti-SARS-CoV-2 antibodies to the general population. Confidence intervals (95% CI) for seroprevalence were estimated using the Clopper-Pearson method. Using logistic regressions, we sought to understand how the IgM and IgG seropositivity are influenced by various variables such as age, gender, occupation, education level, nationality, type of accommodation, etc. The analyses were carried out after carefully handling the database and processing missing data.

### Ethics statement

This research complies with ethical recommendations. The project protocol has been validated by the National Health Research Ethics Committee (CNERS) of the Ministry of Health and Social Action. The research protocol was drawn up following Senegalese laws and regulations governing the confidentiality of personal data. The study was approved by the Senegalese National Ethics Committee for Research in Health (Reference number N°000043/MSAS/CNERS/SP, 28 February 2022).

## Results

### Baseline characteristics of participants

**Fig 1:**
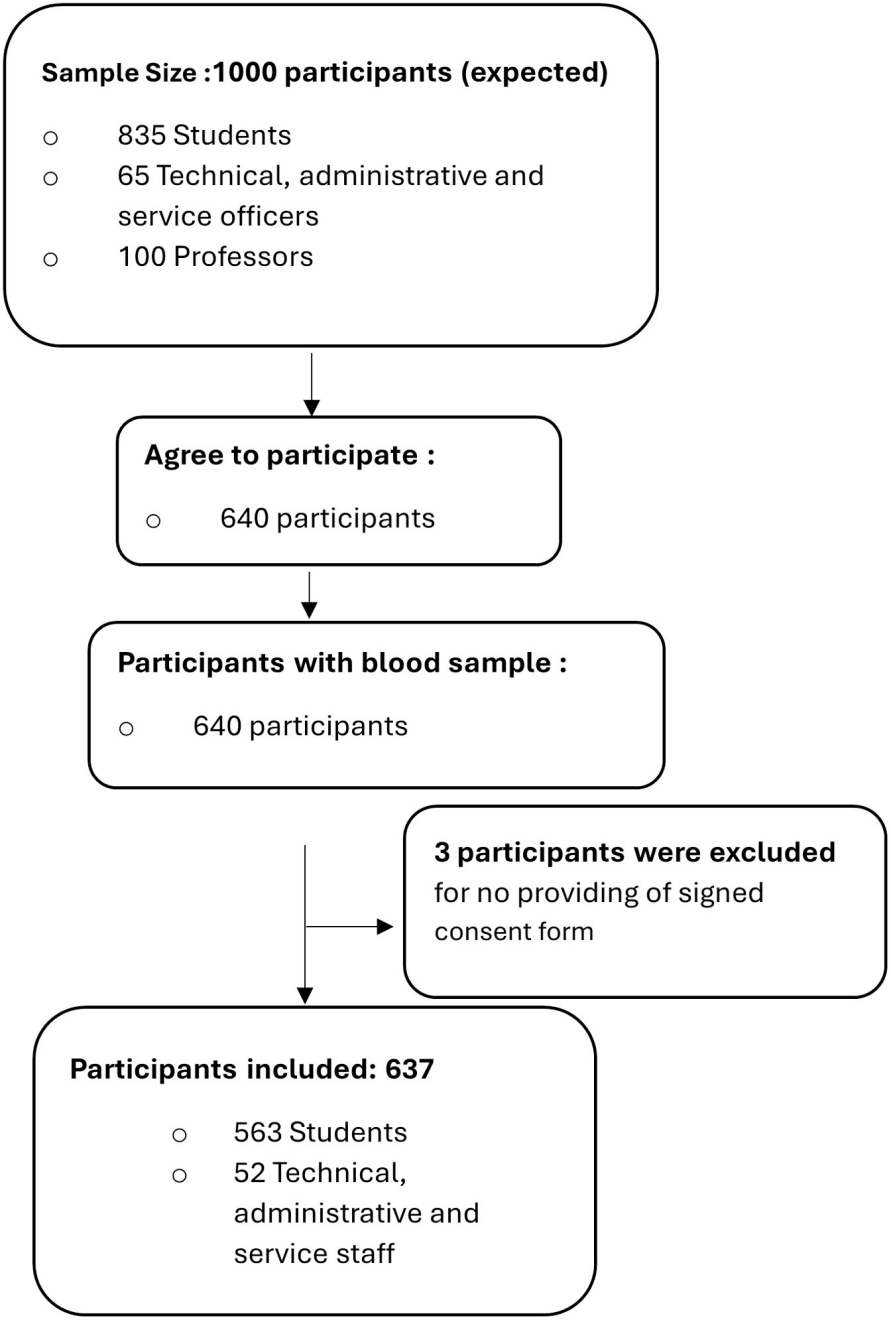
Flowchart of participants’ enrolment for the anti-SARS-CoV-2 antibody seroprevalence study in Superior Polytechnic School from May 19 to August 18, 2022.

From the 1000 students, professors and TAS officers of PHS who were invited to participate in the study between 19 May 2022 and 18 August 2022, 637 (63.7%) participants were finally included (Fig 1). The most represented group was students (88.38%), followed by TAS officers (8.16%), and professors (3.45%). The mean age was 23.20 years, ranging from 18 to 63 years. The population’s median age was 21 years, with most participants aged 18 to 25 years, representing 85.7% of the population (Table 1). 62.0% of the participants were female, and 36.89% were male, with a male-to-female ratio = 0.59. Most participants were Senegalese (94.66%) and lived in family homes (54.6%). In addition, most ethnic groups in the PHS population were Wolof (31.24%), Fula (24.33%) and Serer (21.98%). The prevalences of active smoking and alcohol consumption were relatively low in the PHS community at the moment of the survey (1.9%) and (3.62%), respectively. Calculation of body mass index also revealed the highest prevalence of normal weight (37.68%), followed by underweight (10.8%) and overweight/obesity, 8.32%). The most common blood group is O+ (47.08%). At the time of data collection, unvaccinated *vs*. vaccinated participants were 57.46% and 35.01% respectively.

**Table 1:** Baseline characteristics of the study population.

Variable	Value, N (%)
**Overall**	637 (100)
**Age (years)**	
Mean	23.20
Median	21
Range	18–63
**Age group (years)**	
[18–25]	546 (85.71)
(25–35]	38 (5.96)
(35–45]	26 (4.08)
(45–55]	12 (1.88)
(55–65]	6 (0.94)
**Gender**	
Female	395 (62)
Male	235 (36.89)
**Occupation**	
Students	563 (88.38)
Professors	22 (3.45)
TASO	52 (8.16)
**Level of education**	
Bachelor degree	450 (70.64)
Master degree	117 (18.36)
PhD	20 (3.14)
others	18 (2.82)
**Nationality**	
Senegalese	603 (94.66)
Others †	32 (5.03)
**Ethnic groups**	
Wolof	153 (24.02)
Fula	139 (21.82)
Serer	79 (12.40)
Jola	16 (2.50)
Malinke	17 (2.67)
Soninke	9 (1.41)
Mauri	7 (1.1)
Others †	18 (2.82)
**Accommodation type**	
Halls of residence	138 (21.67)
Family home	479 (75.19)
**Family members**	
**Family members**	
1–2	37 (5.81)
3–5	247 (38.77)
6–8	188 (29.51)
9 or plus	74 (11.61)
**Matrimonial status**	
Single	558 (87.60)
Married	60 (9.42)
Divorced	2 (0.31)
**Smoker**	
Yes	10 (1.88)
No	538 (84.30)
Stopped > 1 year	10 (1.41)
**Alcohol consumer**	
Yes	21 (3.62)
No	554 (86.81)
Stopped > 1 year	3 (0.31)
**BMI Categorized**	
Underweight	69 (10.83)
Normal weight	240 (37.68)
Overweight /Obesity	53 (8.32)
**Blood Group**	
A-	9 (1.41)
A+	140 (21.98)
AB-	1 (0.15)
AB+	25 (3.92)
B-	5 (0.78)
B+	102 (16.01)
O-	24 (3.77)
O+	300 (47.08)
**COVID-19 Vaccination**	
Yes	221 (35.01)
No	365 (57.46)
Prefer not to say	51 (7.54)

N number; BMI Body Mass Indice; TASO technicians, administrative and service officers

^†^ For nationality and ethnic group, others correspond to others of African nationality or ethnic group with very small numbers (maximum 5).

### Seroprevalence of IgM et IgG SARS-CoV-2 antibodies

Out of all participants, 6.91% (95% CI: 4.93–8.87) were seropositive for SARS-CoV-2 IgM antibody, and 92% (95% CI: 89.90–94.11) were seropositive for SARS-CoV-2 IgG (Table 2). Among those who tested positive for IgM, 6.75% were also positive for IgG, and 0.15% were negative for IgG. interestingly, 6.90% of participants tested negative for both IgM and IgG (S1 Fig). Finally, we found that 83.67% were IgM negative and IgG positive. Then, we also analysed IgM and IgG seroprevalence according to the different characteristics of the study population.

**Table 2:** Distribution of SARS-CoV-2 Ig M and G seropositive and seronegative individuals among the 637 participants enrolled.

Variable	IgM			IgG		
	Seronegative N (%)	Seropositive N (%)	*p-value*	Seronegative N (%)	Seropositive N (%)	*p-value*
**Overall**	593 (93.09)	44 (6.91)		51 (8)	576 (92)	
**Age (years)**						
Mean	23.06	25.16		22.4	23.28	
Median	21	21		21	21	
Range	18–63	18–59		18–47	18–63	
**Age group**			**<0.001** *			0,98
[18–25]	516 (94.50)	30 (5.5)		53 (9.7)	493 (92.3)	
(25–35]	29 (76.31)	9 (23.69)		4 (10.53)	34 (89.47)	
(35–45]	24 (92.31)	2 (7.69)		2 (7.69)	24 (92.31)	
(45–55]	12 (100)	0 (0)		1 (8.33)	11 (91.67)	
(55–65]	4 (66.67)	2 (33.34)		0 (0)	6 (100)	
**Gender**			0.16			0.89
Female	373 (94.43)	22 (5.57)		37 (9.36)	358 (90.63)	
Male	213 (90.64)	22 (9.36)		24 (10.21)	211 (89.79)	
**Occupation**			0.15			0.71
Students	528 (93.78)	35 (6.22)		55 (9.77)	508 (90.23)	
Professors	19 (86.36)	3 (13.64)		1 (9.61)	21 (90.38)	
TASO	46 (88.46)	6 (11.54)		5 (4.54)	47 (95.45)	
**Level of education**			0.18			0.45
Bachelor degree	423 (94)	27 (6)		46 (10.22)	404 (89.78)	
Master degree	105 (84.74)	12 (10.26)		12 (10.25)	105 (89.75)	
PhD	17 (85)	3 (15)		1 (5)	19 (95)	
others	16 (88.89)	2 (11.11)		0 (0)	18 (100)	
**Nationality**			0.29			0.30
Senegalese	565 (93.39)	40 (6.61)		56 (9.26)	549 (90.74)	
Others **†**	26 (86.67)	4 (13.33)		5 (16.67)	25 (83.33)	
**Ethnic groups**			0.25			0.17
Wolof	145 (94.77)	8 (5.23)		14 (9.15)	139 (90.85)	
Fula	128 (92.09)	11 (7.91)		11 (7.91)	128 (92.09)	
Serer	72 (91.14)	7 (8.86)		8 (10.13)	71 (89.87)	
Jola	14 (87.50)	2 (12.50)		0 (0)	16 (100)	
Malinke	16 (94.12)	1 (5.88)		3 (17.65)	14 (82.35)	
Soninke	9 (100)	0 (0)		3 (33.33)	6 (66.67)	
Mauri	7 (100)	0 (0)		0 (0)	7 (100)	
**Others †**	14 (77.78)	4 (22.22)		2 (11.12)	16 (88.88)	
**Accommodation type**			0.73			0.76
Halls of residence	127 (92.03)	11 (7.97)		12 (8.69)	126 (91.31)	
Family home	447 (93.32)	32 (6.68)		48 (10.02)	431 (89.98)	
**Family members**			0.54			0.32
1–2	36 (97.30)	1 (2.70)		2 (5.40)	35 (94.60)	
3–5	227 (91.90)	20 (8.10)		29 (11.75)	218 (88.25)	
6–8	176 (93.62)	12 (6.38)		20 (10.64)	168 (89.36)	
9 or plus	78 (90.70)	8 (9.30)		5 (5.81)	81 (94.19)	
**Matrimonial Status**			0.062			0.53
Single	524 (93.9)	34 (6.1)		54 (9.68)	504 (90.32)	
Married	51 (85)	9 (15)		7 (11.67)	53 (88.33)	
Divorced	2 (100)	0 (0)		0 (0)	2 (100)	
**Smoker**			0.08			0.16
Yes	10 (100)	0 (0)		0 (0)	14 (100)	
No	503 (93.49)	35 (6.51)		57 (9.69)	531 (90.31)	
Stopped > 1 year	8 (80)	2 (20)		0 (0)	10 (100)	
**Alcohol consumer**			0.12			**0.038**
Yes	19 (90.48)	2 (9.52)		1 (4.76)	20 (95.24)	
No	516 (93.14)	38 (6.86)		51 (9.20)	503 (90.80)	
Stopped > 1 year	3 (100)	0 (0)		1 (33.34)	2 (66.66)	
**BMI Categorized**			0.089			0.55
Underweight	67 (97.10)	2 (2.90)		6 (8.70)	63 (91.30)	
Normal weight	224 (93.33)	16 (6.67)		28 (11.67)	212 (88.33)	
Overweight /Obesity	46 (86.79)	7 (11.36)		8 (15.09)	45 (84.91)	
**Blood Group**			0.95			0.094
A-	8 (88.89)	1 (11.11)		1 (11.11)	8 (88.89)	
A+	131 (93.57)	9 (6.43)		14 (10)	126 (90)	
AB-	1 (100)	0 (0)		1 (100)	0 (0)	
AB+	24 (96)	1 (4)		3 (12)	22 (88)	
B-	4 (80)	1 (20)		0 (0)	5 (100)	
B+	94 (92.16)	8 (7.84)		7 (6.86)	95 (93.14)	
O-	22 (91.67)	2 (8.33)		1 (4.17)	23 (95.83)	
O+	279 (93)	21 (7)		29 (9.67)	271 (90.33)	
**COVID-19 Vaccination**			0.624			**<0.0001**
Yes	203 (91.85)	18 (8.15)		7 (3.17)	214 (96.83)	
No	340 (95.15)	25 (6.85)		49 (13.42)	316 (86.58)	

*Statistics were calculated without missing values. Missing values were excluded from the analysis

The IgM seroprevalence results showed significant age-dependence (*p* < 0.001) with higher seroprevalence in the (25–35) age group with 23.69% (95% CI: 20.39–27) compared to (18–25) age group with 5.5% (95% CI: 3.73–7.27) (Table 2). According to gender, IgM seroprevalence was higher in men, 9.4% (95% CI: 7.13–11.67) than in women, 5.6% (95% CI: 3.51–7.38), but no significant differences were found by sex (*p* = 0.83). There were no significant differences according to the other sociodemographic parameters regarding IgM antibodies seropositivity, such as occupation, level of education, ethnic group, accommodation type and size, but with some differences between sub-groups. For example, depending on the type of residence, we found a higher IgM seroprevalence among those living in halls of residence, 7.97% (95% CI: 5.87–10.07) compared to those living in family homes, 6.68% (95% CI: 4.74–8.62)], *p* = 0.73.

Regarding IgG seroprevalence, we found significant differences between those who drink alcohol (95.24%, 95% CI: 93.58–96.89), those who don’t (90.80%, 95% CI: 88.55–93.04) and those who stopped drinking more than a year ago (66.66%, 95% CI: 64.35–68.07) with a *p* = 0.038 (Table 2). Additionally, a very significant difference was found based on vaccination status, with a higher seropositivity rate among vaccinated individuals (96.83%, 95% CI: 95.47–98.19) compared to unvaccinated individuals (86.58%, 95% CI: 83.93–89.22), *p* < 0.0001. No significant difference was found concerning IgG seroprevalence across different age groups. In terms of gender, IgG seroprevalence was lower in men (89.9%, 95% CI: 87.60–92.24) compared to women (90.6%, 95% CI: 88.33–92.87), with a p-value of 0.98. No significant differences were found by sex (p = 0.83). Furthermore, while IgM and IgG seroprevalences were higher in professors and TAS officers than students, this difference was not statistically significant (p = 0.71).

### Compliance with preventive measures and antibodies’ seroprevalence

**Fig 2:**
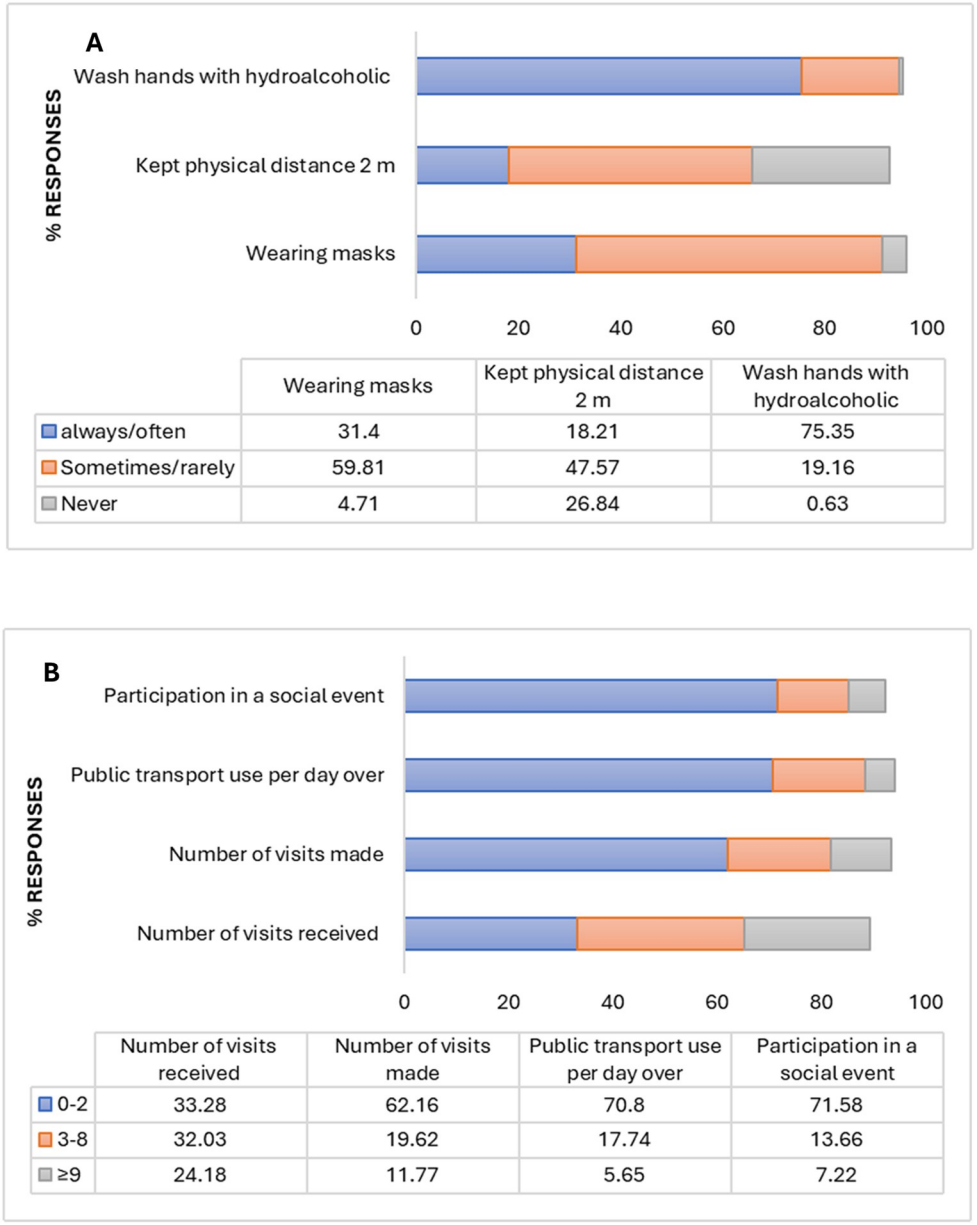
Compliance with preventive measures recommended by authorities. An interviewer-administered questionnaire was distributed to participants, containing questions about compliance with WHO’s recommended preventive measures, and then the rate of responses was determined. **A)** Responses related to the frequency of personal hygiene and physical distancing; and **B)** social distancing in the last 15 days before the survey. Values below bars indicate specific percentages.

Many respondents did not comply with the recommended preventive measures. Only 31.39% reported wearing masks often or always, and 18.21% maintained a physical distance of at least 2 meters over the last 15 days. However, most participants did comply with hand washing, with 75.35% reporting frequent hand washing (Fig 2A). Regarding social distancing, 71.58% reported participating in 0–2 social events, 70.8% used public transport 0–2 times a day, and 62.16% visited someone in the 15 days before the survey (Fig 2B). Antibody seroprevalence revealed significant IgM seropositivity related to the number of visits made to someone in the 15 days before the survey (*p* = 0.008). However, there was no significant difference in IgG seropositivity and compliance with barrier measures (S1 Table).

### Diagnosis and clinical symptoms related to COVID-19 and antibodies’ seroprevalence

**Fig 3:**
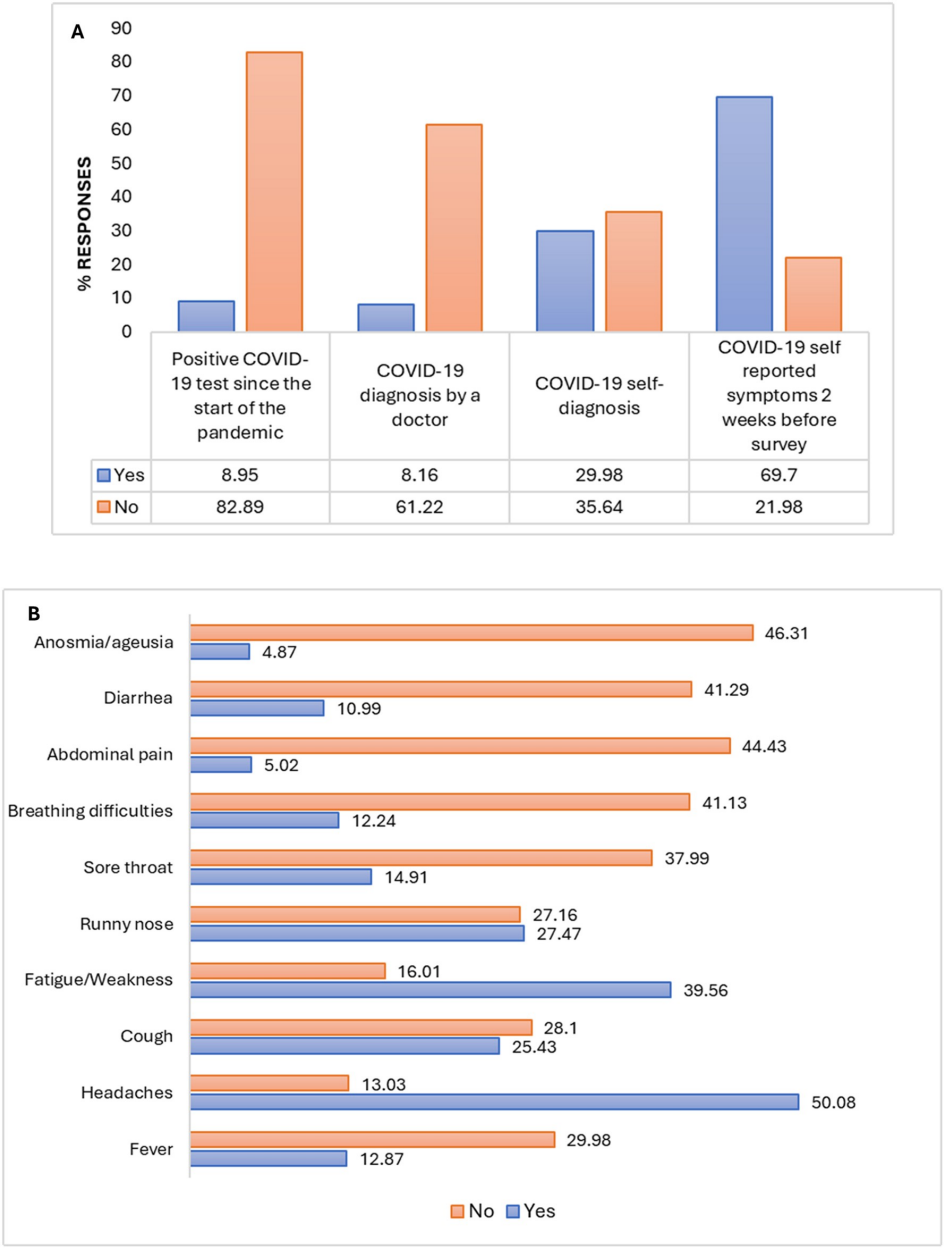
Diagnosis and clinical symptoms related to COVID-19. **A)** Relative frequency distribution (%) of different clinical diagnostics of COVID-19 and; **B)** symptoms of COVID-19, show that most symptoms were headaches, Fatigue or Weakness and runny nose, in respondents of our study. Notes: The values are shown in percentage.

During the survey, a low rate of respondents, 8.95% declared to have tested positive by RT-PCR for COVID-19 infection since the beginning of the pandemic, while 82.89% declared to have tested negative. 8.16% did not disclose their test results (Fig 3A). 8.10% of respondents reported being diagnosed as COVID-19 positive by a healthcare professional, and 29.98% reported self-diagnosing as COVID-19 positive. A significant number of participants (69.70%), declared experiencing symptoms consistent with COVID-19 in the 15 days before the survey, with common symptoms including cough (25.43%), Fatigue/Weakness (39.56%), fever (12.87%), sore throat (14.91%) and headache (50.08%). Finally, 140/637 (21.98%) participants were asymptomatic (Fig 3B).

Concerning the seroprevalence of IgM and IgG antibodies based on COVID-19 diagnosis and clinical symptoms, we found those who did not self-diagnose the disease had a higher IgM seropositivity [10.57% (95% CI: 7.88–12.59)] compared to those who did self-diagnose [5.24% (95% CI: 3.51–6.97)] (Table 3). Regarding clinical symptoms, we found no significant differences in terms of IgM seropositivity, but there was a higher IgG seroprevalence in participants reporting fatigue symptoms [92.06% (95% CI: 89.96–94.16)] compared to those who did not [80.39% (95% CI: 77.31–83.47)] with a significant p = 0.0027.

**Table 3:** Seroprevalence according to diagnosis and clinical symptoms related.

	IgM			IgG		
	Seronegative N (%)	Seropositive N (%)	*p- value*	Seronegative N (%)	Seropositive N (%)	*p- value*
**Positive COVID-19 test since the start of the pandemic**			0.411			0.814
Yes	51 (89.47)	6 (10.53)		6 (10.53)	51 (89.47)	
No	493 (93.37)	35 (6.63)		51 (9.66)	477 (90.34)	
**COVID-19 diagnosis by a doctor**			0.276			0.281
Yes	51 (98.08)	1 (1.92)		8 (15.38)	44 (84.62)	
No	363 (93.08)	27 (6.92)		37 (9.49)	353 (90.51)	
**COVID-19 self-diagnosis**			0.049 *			0.45
Yes	181 (94.76)	10 (5.24)		22 (11.52)	169 (88.48)	
No	203 (89.43)	24 (10.57)		20 (8.81)	207 (91.19)	
**COVID-19 symptoms 2 weeks before survey**			0.871			0.835
Yes	413 (93.02)	31 (6.98)		46 (10.36)	398 (89.64)	
No	129 (92.14)	11 (7.86)		13 (9.29)	127 (90.71)	
**Fever**			1			0.242
Yes	77 (93.90)	5 (6.10)		6 (7.32)	76 (92.68)	
No	178 (93.19)	13 (6.81)		25 (13.09)	166 (86.91)	
**Headaches**			0.727			0.817
Yes	298 (93.42)	21 (6.58)		36 (11.28)	283 (88.72)	
No	76 (91.57)	7 (8.43)		8 (9.64)	75 (90.36)	
**Cough**			0.804			0.509
Yes	150 (92.59)	12 (7.41)		17 (10.49)	145 (89.51)	
No	168 (93.86)	11 (6.14)		24 (13.41)	155 (86.59)	
**Fatigue**			0.372			0.0027 *
Yes	238 (94.44)	14 (5.56)		20 (7.94)	232 (92.06)	
No	93 (91.18)	9 (8.82)		20 (19.61)	82 (80.39)	
**Runny nose**			0.977			0.489
Yes	164 (93.71)	11 (6.29)		19 (10.86)	156 (89.14)	
No	161 (93.06)	12 (6.94)		24 (13.87)	149 (86.13)	
**Sore throat**			0.729			1
Yes	87 (91.58)	8 (8.42)		11 (11.58)	84 (88.42)	
No	226 (93.39)	16 (6.61)		28 (11.57)	214 (88.43)	
Missing	280 (93.33)	20 (6.67)		22 (7.33)	278 (92.67)	
**Breathing difficulties**			0.61			0.23
Yes	74 (94.87)	4 (5.13)		6 (7.69)	72 (92.31)	
No	242 (92.37)	20 (7.63)		35 (13.36)	227 (86.64)	
**Abdominal pain**			0.92			0.39
Yes	30 (93.75)	2 (6.25)		2 (6.25)	30 (93.75)	
No	262 (93.58)	21 (7.42)		38 (13.43)	245 (86.57)	
**Diarrhea**			0.25			0.59
Yes	68 (97.14)	2 (2.86)		7 (10)	63 (90)	
No	243 (92.39)	20 (7.61)		35 (13.31)	228 (86.69)	
**Anosmia/ageusia**			1			0.397
Yes	29 (93.55)	2 (6.45)		2 (6.45)	29 (93.55)	
No	274 (92.88)	21 (7.12)		39 (13.22)	256 (86.78)	

### Risk factors associated with SARS-CoV-2 IgM and IgG seropositivity among PHS community

Multiple Logistic regression analysis didn’t show an association between IgG seropositivity and the factors studied. Interestingly, an association between IgM seropositivity and participants’ ethnic group [O.R. 0.723 95% CI: 0.49–0.96, (*p* = 0.046)] was reported (Table 4). Using a univariate analysis, we found a significant association between IgM seropositivity and marital status [O.R. 2.399 95% CI: 1.08–4.87 (*p* = 0.021)], and Body Mass Indice (BMI) categorised [O.R. 2.015 95% CI: 1.12–3.56, (*p* = 0.016)]. In addition, on one hand, we found a significant association between vaccination and IgG seropositivity [O.R. 4.741 95% CI: 2.25–11.65, (*p* < 0.001)]; on the other hand, no associations were observed between SARS-CoV-2 seropositivity and sex and age groups. Regarding COVID-19 symptoms, we found a significant association between fatigue symptoms and IgG seropositivity [O.R. 2.829, 95% CI: 1.44–5.55 (p = 0.0023)] (Table 5). Finally, no significant association were found between IgM and IgG seropositivity and preventive measures (S2 Table).

**Table 4:** Multiple logistic regression of sociodemographical risk factors affecting seropositivity.

	IgM				IgG			
Variable	Univariate OR (95% CI)	*p- value*	Multivariate OR (95% CI)	*p- value*	Univariate OR (95% CI)	*p-value*	Multivariate OR (95% CI)	*p- value*
**Age**	1.034 [0.99–1.06]	0.055	0.971 [0,74–1,21]	0,808	1.023 [0.98–1.081]	0.345	0.974 [0.89–1.071]	0.573
**Gender**	1.75 [0.943–3.25]	0.073	0.776 [0,10–4.6]	0.791	0.906 [0.53–1.578]	0.728	0.913 [0.52–1.62	0.757
**Occupation**	1.652 [0.92–2.72]	0.065	1.345[0,06–22.03]	0.834	1.26 [0.69–2.75]	0.508	2.545 [0.60–15.33]	0.250
**Level of education**	1.526 [0.91–2.47]	0.095	1.709 [0.31–9.47]	0.532	0.973 [0.61–1.61]	0.911	0.917 [0.48–1.73]	0.790
**Nationality**			0.074 [0.003–2.21]	0.083	1.96 [0.64–4.93]	0.186	2.286 [0.49–7.82]	0.225
**Ethnic groups**	0.985 [0.86–1.13]	0.826	0.723 [0.49–0.96]	0.046 *	0.95 [0.84–1.07]	0.426	0.967 [0.85–1.095]	0.6
**Place of residence**	0.995 [0.72–1.23]	0.740	0.562 [0.17–1.24]	0.23	0.998 [0.80–1.27]	0.992	0.981 [0.74–1.32]	0.894
**Accommodation type**	1.21 [0.57–2.40]	0.60	4.209 [0.91–21.62)	0.067	1.17 [0.62–2.37]	0.643	0.924 [0.41–2.23]	0.854
**Family members**	1.133 [0.77–1.66]	0.522	1.849 [0.78–4.78]	0.175	1.138 [0.81–1.6]	0.452	1.234 [0.83–1.85]	0.296
**Maritus status**	2.399 [1.08–4.87]	0.021 *	4.44 [0.28–54.32]	0.235	1.02 [0.47–2.68]	0.956	1.135 [0.43–3.95]	0.818
**Smoking**	1.004 [0.21–2.48]	0.995	2.897 [0.087–36.8]	0.457				
**Alcohol**	1.064 [0.18–3.17]	0.926	2.06 [0.066–30.84}	0.622	0.835 [0.32–3.25]	0.747		
**BMI Categorized**	2.015 [1.12–3.56]	0.016 *	1.215 [0.39–3.73]	0.729	0.785 [0.49–1.29]	0.329	0.718 [0.43–1.21]	0.206
**Blood Group**	1.011 [0.89–1.152]	0.861	1.049 [0.786–1.47]	0.235	1.023 [0.91–1.14]	0.679	0.988 [0.86–1.24]	0.842
**Vaccination Status**	1.206 [0.63–2.25]	0.560	0.428 [0.08–1.89]	0.285	4.741 [2.25–11.65]	<0.001 *	2.714 [0.59–48.48]	0.325

OR odds ratio. Signifcant values are in bold.

**Table 5:** Multiple logistic regression of diagnosis and clinical symptoms affecting seropositivity.

	IgM		IgG	
Variable	Univariate OR (95% CI)	*p- value*	Univariate OR (95% CI)	*p- value*
**Positive COVID-19 test since the start of the pandemic**	1.657 [0.60–3.87]	0.278	0.909 [0.398–2.45]	0.834
**COVID-19 diagosis by a doctor**	0.264 [0.014–1.28]	0.95	0.576 [0.263–1.40]	0.191
**COVID-19 self-diagosis**	0.467 [0.21–0.97]	0.051	0.742 [0.39–1.41]	0.360
**COVID-19 symptoms 2 weeks before survey**	0.880 [0.44–1.88]	0.727	0.885 [0.45–1.65]	0.713
**Fever**	0.889 [0.28–2.45]	0.829	1.908 [0.80–5.306]	0.174
**Cough**	1.222 [0.52–2.89]	0.643	1.321 [0.68–2.59]	0.409
**Fatigue**	0.608 [0.26–1.50]	0.262	2.829 [1.44–5.55]	0.0023 *
**Headaches**	0.765 [0.33–2]	0.556	0.838 [0.35–1.79]	0.669
**Sore throat**	1.299 [0.51–3.06]	0.5	0.999 [0.488–2.18]	0.998
**Runny nose**	0.889 [0.38–2.11]	0.807	1.323 [0.697–2.54]	0.394
**Breathing difficulties**	0.654 [0.186–1.79]	0.451	1.850 [0.80–5.046]	0.183
**Abdominal pain**	0.831 [0.13–3.03]	0.809	2.327 [0.66–14.74]	0.261
**Diarrhea**	0.357 [0.056–1.27]	0.172	1.382 [0.62–3.52]	0.460
**Anosmia/ageusia**	0.899 [0.14–3.28]	0.890	2.209 [0.63–14.00]	0.291

## Discussion

This study represents the first sero-epidemiological survey of COVID-19 carried out in an educational establishment in Senegal. The survey was carried out on samples collected from personnel and students from May 2022 to August 2022, after the fourth wave of COVID-19 in Senegal. A total of 637 participants were included, with 62% women and 37% men. The median age was 21, justified by the study being conducted in a university environment. Consequently, the study population mainly consisted of students representing approximately 86% of the participants. 35% of the participants have either received partial or full vaccination, which is higher than the national vaccination rate of 16.9% at the time of the survey [33]. This could be attributed to the high level of education and awareness about the importance of vaccination among our population. Additionally, the CAD University authorities tried to set up a vaccination campaign at various sites during October 2021. However, the vaccination rate remains low compared to global levels, with nearly 70% of the population having received at least one vaccine dose since the pandemic’s beginning [34].

The study showed IgG and IgM SARS-CoV-2 antibody seroprevalences of 92% (95% CI: 89.90–94.11) and 6.91% (95% CI: 4.93–8.87), respectively. Interestingly, 6.90% of participants tested negative for both IgM and IgG corresponding to persons who probably lacked antibodies following infection. Indeed, Absence of antibodies varied independently by illness severity, race/ethnicity, obesity, and immunosuppressive drug therapy. The proportion of seronegative remained relatively stable among persons tested up to 90 days post-symptom onset [35]. The IgM seroprevalence could indicate that the virus was circulating within PHS at the time of the survey. Indeed, in seroprevalence surveys, it is recommended to use IgM and IgG detection to track the spread of infection and defining herd immunity barrier and individual immunization levels in the ongoing COVID-19 pandemic [36]. IgM antibody levels rise around a week after the initial infection [22]. IgG antibodies appear later than IgM antibodies (generally within 14 days of infection). They can persist for up to a year [37, 38], meaning that IgG antibodies serve as an indicator of infection or vaccination status [39]. The high IgG seroprevalence in our study may be due to increased SARS-CoV-2 exposure and the COVID-19 vaccination policy. Our study took place after the fourth wave of COVID-19 in Senegal in January 2022, and the vaccine rollout began in February 2021 [40], which could explain our result. The first SARS-CoV-2 Senegalese seroepidemiological surveys in 2020 showed seroprevalence around 20–30% [41, 42]. Since then, studies have indicated a sharp increase in seroprevalence, possibly due to the circulation of various variants, including the deadly delta variant [11, 40, 43, 44]. Similar high seroprevalence rates have been observed in other African countries during the same period, highlighting the importance of conducting regular epidemiological surveys to track the spread of SARS-CoV-2 [45–47].

Our results also showed a different distribution by age group, with lower IgM seroprevalence in younger individuals, particularly those aged 18–25 years, compared to those aged 55–65 years (5.5% and 33.34%, respectively) with a *p* < 0,001. The 18–25 age group appears less affected by recent infections. This reinforces the data showing that young people are less affected by SARS-CoV-2 infection [48]. In Senegal, it was reported during the first wave that the seroprevalence of SARS-CoV-2 was higher in patients older than 65 years [49]. Afterwards, the study investigated IgM seroprevalence based on the type of residence. Surprisingly, there was no significant difference in IgM seroprevalence between those living in halls of residence (7.97%, 95% CI: 5.87–10.07) and those living in family homes (6.68%, 95% CI: 4.74–8.62), with a *p* = 0.73. Our study disproves the hypothesis that the environment at CAD University would create a climate conducive to spreading the virus. The PHS community, primarily made up of young people, does not appear to be contributing to new infections [48, 50].

During the survey, a small percentage of participants (8.95%) reported testing positive for COVID-19 infection through RT-PCR since the beginning of the pandemic. 8.16% were diagnosed as COVID-19 positive, and 29.98% reported having been self-diagnosed as COVID-19 positive. These results, like those of vaccination rate, contrast with the high seroprevalence of IgG, which was over 90% for the study population. In Senegal, limited access to rapid RT-PCR diagnostic tests has hindered COVID-19 detection [51]. WHO reported that only one in seven COVID-19 infections in Africa are being detected, emphasizing the need to expand testing capabilities [52]. Senegal, with a population of 17 million, has conducted about 1.5 million tests, whereas the USA, with a population of around 335 million, has administered over 880 million tests [53].

Approximately 70% of the participants showed symptoms associated with COVID-19 at the time of the survey. The most common symptoms reported were cough, fatigue/weakness, fever, sore throat, and headache. According to China CDC data, individuals with COVID-19 have experienced a wide range of symptoms, varying from mild to severe illness. Symptoms may appear 2–14 days after exposure to the virus. However, these symptoms can be similar to those of the flu caused by influenza [54]. In Senegal, influenza circulates year-round with two peaks: January to March and August to October. However, the expected January–March peak in 2021–2022 disappeared due to active SARS-CoV-2 circulation. An unexpected influenza peak was observed from May to July 2022, suggesting potential viral interference that needs further investigation in tropical settings [55].

In our study, we observed a low level of compliance with the preventive measures recommended by health authorities such as wearing masks (31.40%) and keeping physical distance (17.42%). However, most participants reported a high level of compliance with hand washing (75.35%). Our study found opposing results compared to Kearney et al., who noted significant adherence to COVID-19 prevention measures among Senegalese participants, especially regarding wearing masks and practising good personal hygiene [56]. The variance in our findings may be attributed to the fact that the individuals in our research are younger and less inclined to adhere to preventive protocols [57, 58]. Our findings showed that IgM seropositivity depends on the number of visits to others, indicating a lack of social distancing (p = 0.008). This highlights the importance of following the World Health Organization’s guidelines on extensive social distancing as a non-pharmaceutical way to reduce the spread of infection and related deaths [59]. However, research has indicated that the effectiveness of social distancing measures varies based on demographic, environmental, and economic factors [60].

We investigated the association of sociodemographic, clinical and lifestyle factors, such as BMI, blood type, smoking and alcohol consumption, with seropositivity using logistics. Then, we found an association between BMI categorised and IgM seropositivity (O.R. 0.238, *p* = 0.043). Previous studies have shown that obese individuals (BMI > 30 kg/m²) were significantly more likely to be seropositive. It is uncertain whether the raised seroprevalence in these groups represents a greater risk of SARS-CoV-2 infection. However, obese individuals are known to experience more severe COVID-19 symptoms [61, 62]. Interestingly, a significant association between IgM seropositivity and ethnic group was found (O.R. 0.723, *p* = 0.046), suggesting a difference in susceptibility to SARS-CoV2- depending on race and ethnicity. According to previous findings, it seems there are disparities in the effects of COVID-19 infection among different racial/ethnic groups [63, 64]. Then, understanding the mechanisms for the disparity will help to evaluate the risk for COVID-19 according to ethnicity.

Our findings showed that IgM seropositivity was associated with marital status (unmarried/married) (O.R. 2.39, *p* = 0.021); another study reveals that transmission of SARS-CoV-2 is variable among different people within the home. For instance, the risk for infection was higher between spouses, at 43%, which could be a reflection of transmission through intimacy or longer or more direct exposure [65]. Regarding vaccination status, we found a relationship between vaccination and IgG seroprevalence (O.R. 4.741, *p* < 0.001), showing that people who had received vaccine doses were four times more likely to produce anti-SARS-CoV-2 antibodies than those who had not. It will be interesting to determine the IgG-neutralizing antibodies and to determine antibody levels according to vaccination status and type. Indeed, it has been shown that vaccines from different manufacturers might induce different antibody responses [66].

Some limitations of our study must be considered. By design, we carried out an on-site university population-based cross-sectional study. Thus, the results cannot be extrapolated directly to the general CAD University population. Participants were subjected to recall bias when completing the questionnaires, particularly preventive measures at the time of contact. We also noted a very low level of participation from professors and TAS officers. This constitutes a bias in the statistical calculations. Missing data for certain criteria and non-prefer responders were a limitation of statistics. To date, our study is the only SARS-CoV-2 seroepidemiological survey conducted in a university community in Senegal. Data on the Serological testing performed by measuring IgM and IgG immunoglobulins against the SARS-CoV-2 targeting peak S1 protein separately is a strength of our study. This allowed us to differentiate recent from previous infections. Finally, with our questionnaire, we were able to collect a lot of data, which was useful in calculating associations with risk factors.

## Conclusions

In summary, our analysis of more than 630 subjects from Polytechnic High School community members estimated the extent of exposure to SARS-CoV-2. Our study revealed a high seroprevalence from May to August 2022 following the fourth wave of COVID-19 in Senegal. Our results show that the majority of students and staff have already been exposed to SARS-CoV-2 and confirm the circulation of the virus (SARS-CoV-2) at the time of the survey as shown by the high IgM and IgG seroprevalence. The data show a link between seropositivity and various factors such as age and non-compliance with prevention measures and Diagnosis and Clinical symptoms. These results underline the importance of seroepidemiological surveys to estimate the real impact of the COVID-19 pandemic and the disparities between populations to establish a profile of the transmission dynamics of the virus. In addition, these results may be essential for the CAD university in the event of the emergence of future waves to make appropriate decisions and put in place means of monitoring the evolution of the pandemic after the relaxation of social distancing measures and the implementation of a vaccination schedule at CAD University, which will serve as a basis for other universities in Senegal.

## Supporting information

S1 Fig Distribution of different antibody response profiles in the study population. The vertical axis (y-axis) represents the IgM/IgG SARS-CoV-2 antibody rate. The horizontal axis (x-axis) represents the different profiles of IgM/IgG responses.
(PDF)

S1 Table Distribution of Sars-Cov-2 Ig M and G seropositive and seronegative according to preventive measures. (DOCX)

S2 Table Multiple logistic regression of preventive measures affecting seropositivity. (DOCX)

## References

[1] Asselah T, Durantel D, Pasmant E, Lau G, Schinazi RF. COVID-19: Discovery, diagnostics and drug development. J Hepatol. 2021;74(1):168–84. Epub 20201008. doi: 10.1016/j.jhep.2020.09.031 33038433 PMC7543767

[2] Yesudhas D, Srivastava A, Gromiha MM. COVID-19 outbreak: history, mechanism, transmission, structural studies and therapeutics. Infection. 2021;49(2):199–213. Epub 20200904. doi: 10.1007/s15010-020-01516-2 32886331 PMC7472674

[3] WHO Director-General’s opening remarks at the media briefing on COVID19 -March 2020.

[4] https://www.worldometers.info/coronavirus/ (accessed November 12, 2023).

[5] Mofijur M, Fattah IMR, Alam MA, Islam A, Ong HC, Rahman SMA, et al. Impact of COVID-19 on the social, economic, environmental and energy domains: Lessons learnt from a global pandemic. Sustain Prod Consum. 2021;26:343–59. Epub 20201014. doi: 10.1016/j.spc.2020.10.016 33072833 PMC7556229

[6] Hosseinzadeh P, Zareipour M, Baljani E, Moradali MR. Social Consequences of the COVID-19 Pandemic. A Systematic Review. Invest Educ Enferm. 2022;40(1). doi: 10.17533/udea.iee.v40n1e10 35485623 PMC9052715

[7] Shang Y, Li H, Zhang R. Effects of Pandemic Outbreak on Economies: Evidence From Business History Context. Front Public Health. 2021;9:632043. Epub 20210312. doi: 10.3389/fpubh.2021.632043 33777885 PMC7994505

[8] World Bank Open Data. Data. https://data.worldbank.org/ (2021).

[9] Mok KH. Impact of COVID-19 on Higher Education: Critical Reflections. High Educ Policy. 2022;35(3):563–7. Epub 20220811. doi: 10.1057/s41307-022-00285-x 35971428 PMC9365678

[10] Selvaraj A, Radhin V, Ka N, Benson N, Mathew AJ. Effect of pandemic based online education on teaching and learning system. Int J Educ Dev. 2021;85:102444. Epub 20210608. doi: 10.1016/j.ijedudev.2021.102444 34518732 PMC8426326

[11] https://www.worldometers.info/coronavirus/country/senegal/ (accessed November 12, 2023)

[12] Diallo AI, Faye A, Tine JAD, Ba MF, Gaye I, Bonnet E, et al. Factors associated with the acceptability of government measures to address COVID-19 in Senegal. Rev Epidemiol Sante Publique. 2022;70(3):109–16. Epub 20220331. doi: 10.1016/j.respe.2022.03.123 35491335 PMC8968153

[13] Ba MF, Ridde V, Diallo AI, Tine JAD, Kane B, Gaye I, et al. Acceptability of contact management and care of simple cases of COVID-19 at home: a cross-sectional study in Senegal. Trans R Soc Trop Med Hyg. 2022;116(12):1214–22. doi: 10.1093/trstmh/trac094 36222221 PMC9619604

[14] Nguyen KH, Nguyen K, Mansfield K, Allen JD, Corlin L. Child and adolescent COVID-19 vaccination status and reasons for non-vaccination by parental vaccination status. Public Health. 2022;209:82–9. Epub 20220613. doi: 10.1016/j.puhe.2022.06.002 35870290 PMC9189141

[15] Zhou Y, Rahman MM, Khanam R. The impact of the government response on pandemic control in the long run-A dynamic empirical analysis based on COVID-19. PLoS One. 2022;17(5):e0267232. Epub 20220504. doi: 10.1371/journal.pone.0267232 35507588 PMC9067654

[16] Bedford J, Enria D, Giesecke J, Heymann DL, Ihekweazu C, Kobinger G, et al. Living with the COVID-19 pandemic: act now with the tools we have. Lancet. 2020;396(10259):1314–6. Epub 20201008. doi: 10.1016/S0140-6736(20)32117-6 33038947 PMC7544497

[17] Torretta S, Zuccotti G, Cristofaro V, Ettori J, Solimeno L, Battilocchi L, et al. Diagnosis of SARS-CoV-2 by RT-PCR Using Different Sample Sources: Review of the Literature. Ear Nose Throat J. 2021;100(2_suppl):131S–8S. Epub 20200831. doi: 10.1177/0145561320953231 32865458 PMC7459180

[18] Chung YS, Lee NJ, Woo SH, Kim JM, Kim HM, Jo HJ, et al. Validation of real-time RT-PCR for detection of SARS-CoV-2 in the early stages of the COVID-19 outbreak in the Republic of Korea. Sci Rep. 2021;11(1):14817. Epub 20210720. doi: 10.1038/s41598-021-94196-3 34285290 PMC8292370

[19] Maniruzzaman M, Islam MM, Ali MH, Mukerjee N, Maitra S, Kamal MA, et al. COVID-19 diagnostic methods in developing countries. Environ Sci Pollut Res Int. 2022;29(34):51384–97. Epub 20220527. doi: 10.1007/s11356-022-21041-z 35619009 PMC9135468

[20] Lai CC, Wang JH, Hsueh PR. Population-based seroprevalence surveys of anti-SARS-CoV-2 antibody: An up-to-date review. Int J Infect Dis. 2020;101:314–22. Epub 20201009. doi: 10.1016/j.ijid.2020.10.011 33045429 PMC7546669

[21] Rostami A, Sepidarkish M, Leeflang MMG, Riahi SM, Nourollahpour Shiadeh M, Esfandyari S, et al. SARS-CoV-2 seroprevalence worldwide: a systematic review and meta-analysis. Clin Microbiol Infect. 2021;27(3):331–40. Epub 20201024. doi: 10.1016/j.cmi.2020.10.020 33228974 PMC7584920

[22] Hou H, Wang T, Zhang B, Luo Y, Mao L, Wang F, et al. Detection of IgM and IgG antibodies in patients with coronavirus disease 2019. Clin Transl Immunology. 2020;9(5):e01136. Epub 20200506. doi: 10.1002/cti2.1136 32382418 PMC7202656

[23] Li Q, Chen L, Li F, He A. Long-term evaluation of the seroprevalence of SARS-CoV-2 IgG and IgM antibodies in recovered patients: a meta-analysis. BMC Infect Dis. 2023;23(1):444. Epub 20230701. doi: 10.1186/s12879-023-08425-3 37393304 PMC10314509

[24] Feikin DR, Widdowson MA, Mulholland K. Estimating the Percentage of a Population Infected with SARS-CoV-2 Using the Number of Reported Deaths: A Policy Planning Tool. Pathogens. 2020;9(10). Epub 20201013. doi: 10.3390/pathogens9100838 33066231 PMC7602107

[25] Cascini F, Failla G, Gobbi C, Pallini E, Hui J, Luxi W, et al. A cross-country comparison of Covid-19 containment measures and their effects on the epidemic curves. BMC Public Health. 2022;22(1):1765. Epub 20220917. doi: 10.1186/s12889-022-14088-7 36115936 PMC9482299

[26] Vusirikala A, Whitaker H, Jones S, Tessier E, Borrow R, Linley E, et al. Seroprevalence of SARS-CoV-2 antibodies in university students: Cross-sectional study, December 2020, England. J Infect. 2021;83(1):104–11. Epub 20210429. doi: 10.1016/j.jinf.2021.04.028 33933527 PMC8081745

[27] Tsitsilonis OE, Paraskevis D, Lianidou E, Pierros V, Akalestos A, Kastritis E, et al. Seroprevalence of Antibodies against SARS-CoV-2 among the Personnel and Students of the National and Kapodistrian University of Athens, Greece: A Preliminary Report. Life (Basel). 2020;10(9). Epub 20200921. doi: 10.3390/life10090214 32967110 PMC7555935

[28] Arnold CRK, Srinivasan S, Rodriguez S, Rydzak N, Herzog CM, Gontu A, et al. SARS-CoV-2 Seroprevalence in a University Community: A Longitudinal Study of the Impact of Student Return to Campus on Infection Risk Among Community Members. medRxiv. 2021. Epub 20210917. doi: 10.1101/2021.02.17.21251942 33619497 PMC7899462

[29] Migliara G, Renzi E, Baccolini V, Cerri A, Donia P, Massimi A, et al. Predictors of SARS-CoV-2 Infection in University Students: A Case-Control Study. Int J Environ Res Public Health. 2022;19(21). Epub 20221103. doi: 10.3390/ijerph192114376 36361257 PMC9655450

[30] Denning DW, Kilcoyne A, Ucer C. Non-infectious status indicated by detectable IgG antibody to SARS-CoV-2. Br Dent J. 2020;229(8):521–4. Epub 20201023. doi: 10.1038/s41415-020-2228-9 33097885 PMC7582419

[31] Assaid N, Arich S, Charoute H, Akarid K, Anouar Sadat M, Maaroufi A, et al. Kinetics of SARS-CoV-2 IgM and IgG Antibodies 3 Months after COVID-19 Onset in Moroccan Patients. Am J Trop Med Hyg. 2023;108(1):145–54. Epub 20221212. doi: 10.4269/ajtmh.22-0448 36509045 PMC9833093

[32] GeurtsvanKessel CH, Okba NMA, Igloi Z, Bogers S, Embregts CWE, Laksono BM, et al. An evaluation of COVID-19 serological assays informs future diagnostics and exposure assessment. Nat Commun. 2020;11(1):3436. Epub 20200706. doi: 10.1038/s41467-020-17317-y 32632160 PMC7338506

[33] Singhal S, Prakash N. A narrative inquiry into the meaning of career identity of Indian emerging adults amid Covid-19. Int J Educ Vocat Guid. 2023:1–26. Epub 20230316. doi: 10.1007/s10775-023-09590-2 37360271 PMC10019401

[34] Garg T, Votta CM, Prakash N, Deldin PJ. An investigation into the effectiveness of Mood Lifters in the context of trauma exposure. Psychol Serv. 2023. Epub 20230824. doi: 10.1037/ser0000789 . 37616078

[35] Petersen LR, Sami S, Vuong N, Pathela P, Weiss D, Morgenthau BM, et al. Lack of Antibodies to Severe Acute Respiratory Syndrome Coronavirus 2 (SARS-CoV-2) in a Large Cohort of Previously Infected Persons. Clin Infect Dis. 2021;73(9):e3066–e73. doi: 10.1093/cid/ciaa1685 33147319 PMC7665429

[36] Wajnberg A, Mansour M, Leven E, Bouvier NM, Patel G, Firpo-Betancourt A, et al. Humoral response and PCR positivity in patients with COVID-19 in the New York City region, USA: an observational study. Lancet Microbe. 2020;1(7):e283–e9. Epub 20200925. doi: 10.1016/S2666-5247(20)30120-8 33015652 PMC7518831

[37] Ahmad A, Haq M, Rehman A, Haq NU. Anti-nucleocapsid IgG antibodies in SARS-CoV-2 recovered health care workers: One year follow-up study. Int J Immunopathol Pharmacol. 2023;37:3946320231187744. doi: 10.1177/03946320231187744 37394899 PMC10328170

[38] Haveri A, Ekstrom N, Solastie A, Virta C, Osterlund P, Isosaari E, et al. Persistence of neutralizing antibodies a year after SARS-CoV-2 infection in humans. Eur J Immunol. 2021;51(12):3202–13. Epub 20211008. doi: 10.1002/eji.202149535 34580856 PMC8646652

[39] Sun B, Feng Y, Mo X, Zheng P, Wang Q, Li P, et al. Kinetics of SARS-CoV-2 specific IgM and IgG responses in COVID-19 patients. Emerg Microbes Infect. 2020;9(1):940–8. doi: 10.1080/22221751.2020.1762515 32357808 PMC7273175

[40] SMHSA (Senegalese Ministry of Health and Social Action) 2022 [Final report of the 2nd seroprevalence survey of Covid-19 in Senegal, 2022.

[41] Ahouidi AD, Anderson M, Diedhiou CK, Dia A, Mbow M, Dia Y, et al. Seroprevalence of SARS-CoV-2 IgG antibodies in a healthcare setting during the first pandemic wave in Senegal. IJID Reg. 2022;2:96–8. Epub 20211224. doi: 10.1016/j.ijregi.2021.12.008 35721431 PMC8702669

[42] Talla C, Loucoubar C, Roka JL, Barry MA, Ndiaye S, Diarra M, et al. Seroprevalence of anti-SARS-CoV-2 antibodies in Senegal: a national population-based cross-sectional survey, between October and November 2020. IJID Reg. 2022;3:117–25. Epub 20220305. doi: 10.1016/j.ijregi.2022.02.007 35720135 PMC8897837

[43] E K, S P, R G, R KL, A B, G M, et al. Advantages and pitfalls in utilizing artificial intelligence for crafting medical examinations: a medical education pilot study with GPT-4. BMC Med Educ. 2023;23(1):772. Epub 20231017. doi: 10.1186/s12909-023-04752-w 37848913 PMC10580534

[44] John B, Poorna TA, E KJ, Philip G, Bharathi A. Infralingular Versus Supralingular Medial Osteotomy in Sagittal Split Osteotomy of the Mandible: A Randomized Control Study. J Oral Maxillofac Surg. 2023;81(3):280–6. Epub 20221229. doi: 10.1016/j.joms.2022.12.008 . 36587932

[45] Ndiaye AJS, Beye M, Lo G, Kacel I, Sow A, Leye N, et al. Genomic Epidemiology of SARS-CoV-2 in Urban Settings in Senegal. Viruses. 2023;15(6). Epub 20230524. doi: 10.3390/v15061233 37376533 PMC10302768

[46] Akanmu S, Herrera BB, Chaplin B, Ogunsola S, Osibogun A, Onawoga F, et al. High SARS-CoV-2 seroprevalence in Lagos, Nigeria with robust antibody and cellular immune responses. J Clin Virol Plus. 2023;3(3):100156. Epub 20230624. doi: 10.1016/j.jcvp.2023.100156 37388808 PMC10289822

[47] Manirakiza A, Malaka C, Longo JD, Yambiyo BM, Diemer SH, Namsenei J, et al. Sero-prevalence of anti-SARS-CoV-2 antibodies among communities between July and August 2022 in Bangui, Central African Republic. J Public Health Afr. 2023;14(8):2315. Epub 20230605. doi: 10.4081/jphia.2023.2315 37753434 PMC10519115

[48] Felsenstein S, Hedrich CM. SARS-CoV-2 infections in children and young people. Clin Immunol. 2020;220:108588. Epub 20200906. doi: 10.1016/j.clim.2020.108588 32905851 PMC7474910

[49] Seck SM, Mbow M, Kane Y, Cisse MM, Faye G, Kama A, et al. Prevalence of SARS-CoV-2 antibodies in hemodialysis patients in Senegal: a multicenter cross-sectional study. BMC Nephrol. 2021;22(1):384. Epub 20211117. doi: 10.1186/s12882-021-02582-w 34789170 PMC8595275

[50] Lu X, Zhang L, Du H, Zhang J, Li YY, Qu J, et al. SARS-CoV-2 Infection in Children. N Engl J Med. 2020;382(17):1663–5. Epub 20200318. doi: 10.1056/NEJMc2005073 32187458 PMC7121177

[51] https://dirlabosn.com/wp-content/uploads/2022/10/LBM-agree-VF-1.pdf (accessed November 12, 2023).

[52] Adepoju P. Closing Africa’s wide COVID-19 testing and vaccination gaps. Lancet Microbe. 2021;2(11):e573. Epub 20211102. doi: 10.1016/S2666-5247(21)00284-6 34934998 PMC8678665

[53] https://covid.cdc.gov/covid-data-tracker/#vaccinations_vacc-people-booster-percent-pop5 (accessed November 12, 2023).

[54] https://www.cdc.gov/flu/symptoms/flu-vs-covid19.htm (accessed November 12, 2023)

[55] Lampros A, Talla C, Diarra M, Tall B, Sagne S, Diallo MK, et al. Shifting Patterns of Influenza Circulation during the COVID-19 Pandemic, Senegal. Emerg Infect Dis. 2023;29(9):1808–17. doi: 10.3201/eid2909.230307 37610149 PMC10461650

[56] Kearney M, Bornstein M, Fall M, Nianogo R, Glik D, Massey P. Cross-sectional study of COVID-19 knowledge, beliefs and prevention behaviours among adults in Senegal. BMJ Open. 2022;12(5):e057914. Epub 20220526. doi: 10.1136/bmjopen-2021-057914 35618332 PMC9136694

[57] Jaureguizar J, Redondo I, Galende N, Ozamiz N. Factors related to compliance with the COVID-19 health regulations among young people. World J Psychiatry. 2021;11(12):1247–58. Epub 20211219. doi: 10.5498/wjp.v11.i12.1247 35070774 PMC8717041

[58] Kim JK, Crimmins EM. How does age affect personal and social reactions to COVID-19: Results from the national Understanding America Study. PLoS One. 2020;15(11):e0241950. Epub 20201110. doi: 10.1371/journal.pone.0241950 33170903 PMC7654776

[59] Yoo TK, Oh E, Kim HK, Ryu IH, Lee IS, Kim JS, et al. Deep learning-based smart speaker to confirm surgical sites for cataract surgeries: A pilot study. PLoS One. 2020;15(4):e0231322. Epub 20200409. doi: 10.1371/journal.pone.0231322 32271836 PMC7144990

[60] Prakash N, Srivastava B, Singh S, Sharma S, Jain S. Effectiveness of social distancing interventions in containing COVID-19 incidence: International evidence using Kalman filter. Econ Hum Biol. 2022;44:101091. Epub 20211202. doi: 10.1016/j.ehb.2021.101091 34894622 PMC8638209

[61] Wolff D, Nee S, Hickey NS, Marschollek M. Risk factors for Covid-19 severity and fatality: a structured literature review. Infection. 2021;49(1):15–28. Epub 20200828. doi: 10.1007/s15010-020-01509-1 32860214 PMC7453858

[62] Aburto S, Cisterna M, Acuna J, Ruiz C, Viscardi S, Marquez JL, et al. Obesity as a Risk Factor for Severe COVID-19 in Hospitalized Patients: Epidemiology and Potential Mechanisms. Healthcare (Basel). 2022;10(10). Epub 20220922. doi: 10.3390/healthcare10101838 36292285 PMC9601462

[63] Mackey K, Ayers CK, Kondo KK, Saha S, Advani SM, Young S, et al. Racial and Ethnic Disparities in COVID-19-Related Infections, Hospitalizations, and Deaths: A Systematic Review. Ann Intern Med. 2021;174(3):362–73. Epub 20201201. doi: 10.7326/M20-6306 33253040 PMC7772883

[64] Carethers JM. Insights into disparities observed with COVID-19. J Intern Med. 2021;289(4):463–73. Epub 20201206. doi: 10.1111/joim.13199 33164230 PMC9325576

[65] Fogh K, Eriksen ARR, Hasselbalch RB, Kristensen ES, Bundgaard H, Nielsen SD, et al. Seroprevalence of SARS-CoV-2 antibodies in social housing areas in Denmark. BMC Infect Dis. 2022;22(1):143. Epub 20220210. doi: 10.1186/s12879-022-07102-1 35144550 PMC8830972

[66] Megasari NLA, Yamani LN, Juniastuti J, Lusida MI, Mori Y. Seroprevalence of SARS-CoV-2 anti-spike IgG antibody among COVID-19 vaccinated individuals residing in Surabaya, East Java, Indonesia. PeerJ. 2023;11:e16142. Epub 20230925. doi: 10.7717/peerj.16142 37780375 PMC10538276

