# Supplementary figures and images for: Serological survey in a university community after the fourth wave of COVID-19 in Senegal
Source: PLoS One. 2024 Nov 21;19(11):e0298509. doi: 10.1371/journal.pone.0298509 (PMC11581233; doi:10.1371/journal.pone.0298509)

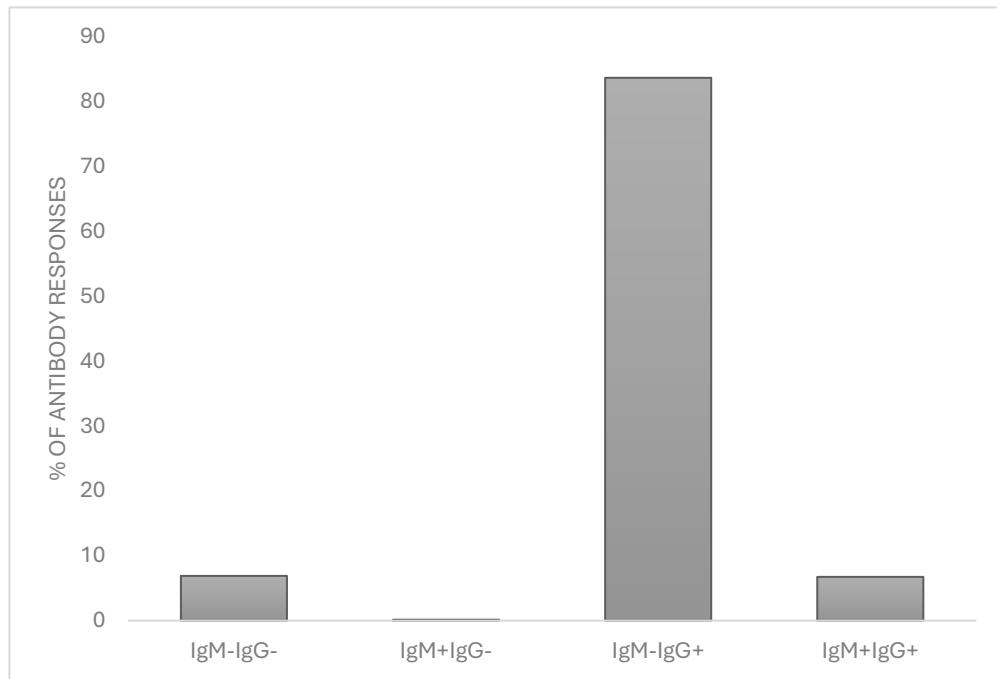

Supplement: S1 Fig — The vertical axis (y-axis) represents the IgM/IgG SARS-CoV-2 antibody rate. The horizontal axis (x-axis) represents the different profiles of IgM/IgG responses. (PDF) [file pone.0298509.s001.pdf]
