# Supplementary material for: Serological survey in a university community after the fourth wave of COVID-19 in Senegal
Source: PLoS One. 2024 Nov 21;19(11):e0298509. doi: 10.1371/journal.pone.0298509 (PMC11581233; doi:10.1371/journal.pone.0298509)
Supplement: S1 Table — (DOCX) [file pone.0298509.s002.docx]

**S1 Table.** Distribution of Sars-Cov-2 Ig M and G seropositive and seronegative according to preventive measures

|  | IgM | | | IgG | | |
| --- | --- | --- | --- | --- | --- | --- |
|  | **Seronegative**  **N ( %)** | **Seropositive**  **N ( %)** | ***p- value*** | **Seronegative**  **N ( %)** | **Seropositive**  **N ( %)** | ***p- value*** |
| Wearing masks |  |  | 0.17 |  |  | 0.73 |
| Always/Often | 183 (91.04) | 18 (8.96) |  | 19 (9.45) | 182 (90.55) |  |
| Sometimes/Rarely | 355 (93.42) | 25 (6.58) |  | 38 (10) | 342 (90) |  |
| Never | 30 (100) | 0 (0) |  | 4 (13.33) | 26 (86.67) |  |
| Kept a physical distance of 2 m |  |  | 0.35 |  |  | 0.33 |
| Always/Often | 110 (94.83) | 6 (5.17) |  | 8 (6.90) | 108 (93.10) |  |
| Sometimes/Rarely | 277 (91.42) | 26 (8.58) |  | 31 (10.23) | 272 (89.77) |  |
| Never | 161 (94.15) | 10 (5.85) |  | 21 (12.28) | 150 (87.72) |  |
| Wash hands with hydroalcoholic  or with soap and water |  |  | 0.105 |  |  | 0.599 |
| Always/Often | 451 (93.96) | 29 (6.04) |  | 51 (10.62) | 429 (89.38) |  |
| Sometimes/Rarely | 108 (88.52) | 14 (11.48) |  | 10 (8.20) | 112 (91.80) |  |
| Never | 4 (100) | 0 (0) |  | 0 (0) | 4 (100) |  |
| Number of visits received in the last 15 days |  |  | 0.583 |  |  | 0.971 |
| 0-2 | 205 (93.61) | 14 (6.39) |  | 23 (10.50) | 196 (89.50) |  |
| 3-8 | 188 (92.16) | 16 (7.84) |  | 21 (10.29) | 183 (89.71) |  |
| ≥9 | 146 (94.80) | 8 (5.20) |  | 15 (9.74) | 139 (90.26) |  |
| Number of visits made in the last 15 days |  |  | **0.008*** |  |  | 0.946 |
| 0-2 | 373 (94.20) | 23 (5.81) |  | 41 (10.35) | 355 (89.65) |  |
| 3-8 | 109 (87.20) | 16 (12.80) |  | 12 (9.60) | 113 (90.40) |  |
| ≥9 | 73 (97.33) | 2 (2.67) |  | 7 (9.33) | 68 (90.67) |  |
| Public transport use per day over the last 15 days |  |  | 0.878 |  |  | 0.668 |
| 0-2 | 419 (92.9) | 32 (7.1) |  | 44 (9.75) | 407 (90.25) |  |
| 3-5 | 105 (92.92) | 8 (7.1) |  | 14 (12.39) | 99 (87.61) |  |
| ≥9 | 33 (91.67) | 3 (8.33) |  | 3 (8.33) | 33 (91.67) |  |
| Participation in a social event in the last 15 days |  |  | 0.589 |  |  | 0.289 |
| 0-2 | 423 (92.76) | 33 (7.24) |  | 51 (11.18) | 405 (88.82) |  |
| 3-8 | 79 (90.80) | 8 (9.20) |  | 5 (5.75) | 82 (94.25) |  |
| ≥9 | 44 (95.65) | 2 (4.35) |  | 4 (8.70) | 42 (91.30) |  |

Significant values are in bold.
