# Supplementary material for: Serological survey in a university community after the fourth wave of COVID-19 in Senegal
Source: PLoS One. 2024 Nov 21;19(11):e0298509. doi: 10.1371/journal.pone.0298509 (PMC11581233; doi:10.1371/journal.pone.0298509)
Supplement: S2 Table — (DOCX) [file pone.0298509.s003.docx]

**S2 Table.** Multiple logistic regression of preventive measures affecting seropositivity

|  | **IgM** | | **IgG** | |
| --- | --- | --- | --- | --- |
| **Variable** | **Univariate**  **OR (95% CI)** | ***p- value*** | **Univariate**  **OR (95% CI)** | ***p- value*** |
| **Wearing masks** | 0.796 [0,5893 - 1,073] | 0.134 | 0.966 [0.74 – 1.25] | 0.793 |
| **Failure to respect the 2m distance** | 0.980 [0,7380 - 1,278] | 0.886 | 1.229 [0.96 – 1.59] | 0.108 |
| **Number of visits received in the last 14 days** | 0.901 [0.701 – 1.15] | 0.404 | 1.002 [0.82 – 1.23] | 0.984 |
| **Number of visits made in the last 14 days** |  |  |  |  |
| **Wash hands with hydroalcoholic** | 0.854 [0.62 – 1.17] | 0.327 | 0.891 [0.68 – 1.17] | 0.405 |
| **Public transport use per day over the last 15 days** | 1.043 [0.75 – 1.40] | 0.793 | 1.072 [0.82 – 1.441] | 0.630 |
| **Participation in a social event in the last 15 days** | 0.952 [0.71 – 1.24] | 0.734 | 1.094 [0.86 – 1.420] | 0.473 |
